# Cytostatic *versus* cytocidal profiling of quinoline drug combinations via modified fixed-ratio isobologram analysis

**DOI:** 10.1186/1475-2875-12-332

**Published:** 2013-09-18

**Authors:** Alexander P Gorka, Lauren M Jacobs, Paul D Roepe

**Affiliations:** 1 Department of Chemistry, Department of Biochemistry, Cellular, & Molecular Biology, and Center for Infectious Disease, Georgetown University, 37th and 'O’ Sts. NW, Washington, DC, 20057, USA; 2 Present address: Chemical Biology Laboratory, National Cancer Institute, 376 Boyles Street, Frederick, MD, 21702, USA

**Keywords:** Isobologram, Combination therapy, Quinoline, Cytocidal, *P. falciparum*

## Abstract

**Background:**

Drug combination therapy is the frontline of malaria treatment. There is an ever-accelerating need for new, efficacious combination therapies active against drug resistant malaria. Proven drugs already in the treatment pipeline, such as the quinolines, are important components of current combination therapy and also present an attractive test bank for rapid development of new concepts.

**Methods:**

The efficacy of several drug combinations versus chloroquine-sensitive and chloroquine-resistant strains was measured using both cytostatic and cytocidal potency assays.

**Conclusions:**

These screens identify quinoline and non-quinoline pairs that exhibit synergy, additivity, or antagonism using the fixed-ratio isobologram method and find tafenoquine – methylene blue combination to be the most synergistic. Also, interestingly, for selected pairs, additivity, synergy, or antagonism defined by quantifying IC_50_ (cytostatic potency) does not necessarily predict similar behaviour when potency is defined by LD_50_ (cytocidal potency). These data further support an evolving new model for quinoline anti-malarials, wherein haem and haemozoin are the principle target for cytostatic activity, but may not be the only target relevant for cytocidal activity.

## Background

Malaria remains a very serious threat to global health, with 300–500 million clinical cases and nearly 1 million deaths annually [1]. Given the growing spread of resistance to current anti-malarial drugs and lack of an effective vaccine [1-4], development of novel, cost-effective, and efficacious drugs is of utmost importance. Decades of use of drugs such as chloroquine (CQ) have rendered several cost-effective quinoline monotherapies ineffective in various malaria endemic regions. For these reasons and others, the WHO currently recommends combination therapies, particularly artemisinin (ART)-based combination therapy (ACT), as first-line treatment for uncomplicated *Plasmodium falciparum*[5]. Currently valuable ACT consists of ART or derivatives, such as dihydroartemisinin, artemether, and artesunate, in combination with longer half-life quinolines, such as amodiaquine (AQ), mefloquine (MQ), or quinine (QN), antibiotics such as doxycycline and clindamycin, or the blood schizonticide lumefantrine [2,4,5]. Addition of a single dose of the 8-aminoquinoline primaquine (PQ) to ACT is also recommended given its transmission blocking and gametocytocidal properties [5-7].

PQ was initially developed as a safer and more effective 8-aminoquinoline alternative to plasmoquine, and is the only member of that drug class currently in use [6,7], although clinical trials with the related drug tafenoquine (TQ) are ongoing. PQ exhibits asexual stage activity against *Plasmodium vivax*[8], kills hypnozoites of *P. vivax* and *Plasmodium ovale*[9,10], and is active against late-stage gametocytes of *P. falciparum*[6,7,11-13]. Studies have consistently shown that PQ can block transmission prior to the number of gametocytes being reduced [14-16]. PQ has poor growth inhibitory activity against intraerythrocytic stages of *P. falciparum*[17,18], likely owing to poor inhibition of haemozoin (Hz) formation *in vitro*[19-23] and an inability to bind ferriprotoporphyrin IX (FPIX) haem in solution [20].

Use of PQ is limited by short plasma half-life and toxicity, principally haemolysis in patients with X-linked glucose-6-phosphate dehydrogenase (G6PD) deficiency [6,7], which varies between 3% and 30% in endemic areas [24]. Recent evidence has suggested that risk of PQ administration in high G6PD-deficient populations might be ameliorated by lower dosing, without loss of gametocytocidal potency [25,26], which might be due to PQ rapidly metabolizing to carboxyPQ, with plasma concentrations of the metabolite 10-fold greater than PQ 4 h post-dosing [27]. Tafenoquine (TQ) (WR238605), a 5-phenoxyl derivative of PQ [28], has improved asexual stage activity [29], a significantly elongated plasma half-life (two weeks *vs* 6 h) [27,30], and a greater therapeutic index [31]. The compound remains haemolytic in G6PD-deficient individuals, though to a lesser extent than PQ [7].

In addition to use with current ACT, coadministration of 8-aminoquinolines with blood schizonticides like CQ and QN have been explored for their anti-relapse and toxicity-reducing effects (see [32] for review). The latter may be due to competition between the 8-aminoquinoline and the coadministered agent for cytochrome P450s, oxidases, or other metabolic enzymes [32]. Bray *et al.* reported that PQ and TQ act synergistically with CQ *in vitro* against the CQ resistant (CQR) K1 strain, with no effect observed against the CQ sensitive (CQS) D10 strain [33], and that PQ analogues lacking the aminoalkyl side chain of PQ do not synergize with CQ. While this synergistic effect has not been quantified clinically or *in vivo*, there is some preliminary evidence that CQ combined with PQ can lead to decreased treatment failure rates against uncomplicated CQR *P. falciparum*[34,35]. PQ-CQ synergy has also been proposed in treatment of CQR cases of *P. vivax* in Thailand [8,36].

The thiazine dye methylene blue (MB) was the first synthetic compound ever used in the treatment of disease [37], and there has recently been a resurgence of interest in the anti-malarial properties of MB. The compound appears to have transmission-blocking effects via potent clearance of late-stage gametocytes [38], high blood schizonticidal activity [38-40], and Hz inhibition potential [41,42], presumably due to accumulation in the digestive vacuole (DV) [38,42] and/or redox-cycling potential [41,43-45]. MB has been observed to inhibit parasite glutathione reductase (GR) [46,47], which could in theory enhance activity of drugs such as CQ that cause oxidative damage (see also [48]). However, Akoachere *et al.* demonstrated that CQ and MB act antagonistically against 3D7 (CQS) and K1 (CQR) strains [39]. Garavito *et al.* later reported this combination to be additive against strain FcM29 (CQR) [40]. Antagonism has also been reported in the field [49,50], however, combination use with ARTs and AQ have proven effective [50,51]. Regardless, in *in vitro* studies, efficacy of these and other drug combinations has been via standard *P. falciparum* growth inhibition assays. No previous studies of drug combination effects have explicitly quantified potency in terms of cytocidal (parasite cell killing) activity.

**Figure 1:**
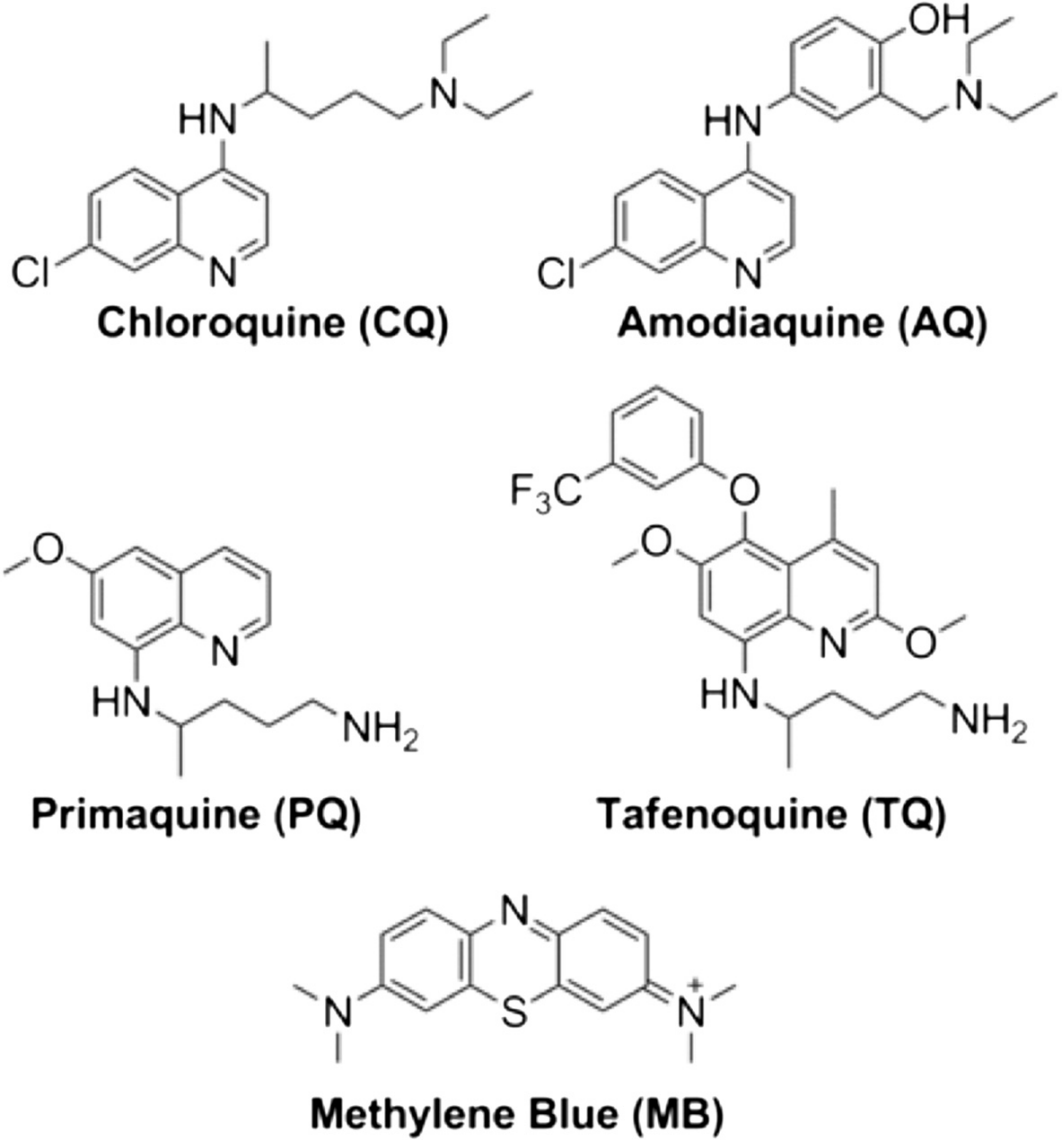
Structures of anti-malarial drugs used in this study.

Given the importance of combination therapy and the attractiveness of further development of combinations using proven, cost-effective, and widely-available drugs like CQ, PQ, and MB (Figure 1), the effects of potentially interesting drug combinations on *in vitro* antiplasmodial cytostatic and cytocidal activity are investigated for both a CQS and CQR strain of *P. falciparum*. Use of isobologram-based approaches (see Methods) resolves conflicting accounts of synergy, additivity, and antagonism [33,39,40], and probes the relationships between these parameters at cytostatic *vs* cytocidal dosages [19,52]. The results suggest an important new concept relevant for the discovery of efficacious combination therapies.

## Methods

### Materials and chemicals

Routine chemicals, media, and solvents were reagent grade or better, purchased from Sigma-Aldrich (St. Louis, MO, USA) or Fisher Scientific (Newark, DE, USA), and used without further purification, unless otherwise noted. Sterile black flat bottom tissue culture, or non-sterile clear polystyrene 96-well plates, and all other laboratory plastics were from Fisher Scientific (Newark, DE). SYBR Green I nucleic acid stain was from Invitrogen (Eugene, OR, USA). Anti-malarial drugs AQ dihydrochloride dihydrate, CQ diphosphate, MB hydrate, PQ bisphosphate, and TQ succinate were from Sigma-Aldrich (St. Louis, MO, USA).

### Parasite culture

*Plasmodium falciparum* clones HB3 (Honduras, CQS), Dd2 (Indochina, CQR), and FCB (Thailand/S. Africa) were obtained from the Malaria Research and Reference Reagent Resource Center (Manassass, VA, USA). Off-the-clot, heat-inactivated pooled type O^+^ human serum and type O^+^ human whole blood were purchased from Biochemed Services (Winchester, VA, USA). Custom 5% O_2_/5% CO_2_/90% N_2_ gas blend was purchased from Robert’s Oxygen (Rockville, MD, USA).

*Plasmodium falciparum* strains were maintained using the method of Trager and Jensen [53] with minor modifications. Briefly, cultures were maintained under an atmosphere containing 5% CO_2_, 5% O_2_, and 90% N_2_ gaseous mix at 2% haematocrit and 1-2% parasitaemia in RPMI 1640 supplemented with 10% type O^+^ human serum, 25 mM HEPES (pH 7.4), 23 mM NaHCO_3_, 11 mM glucose, 0.75 mM hypoxanthine, and 20 μg/L gentamicin, with regular media changes every 48 h.

### Anti-plasmodial assays

Anti-plasmodial cytostatic (growth inhibitory, quantified by the half-maximal growth inhibitory dose or IC_50_) and cytocidal (cell killing, quantified by the half-maximal lethal dose or LD_50_) activity was assessed against the above strains as previously described [52,54], with minor modifications. The cytocidal assay utilizes a 6 h bolus dose with high concentrations of drug followed by washing drug away and growth in the absence of drug for 48 h, while the cytostatic assay utilizes continuous growth for 48 h in the constant presence of low concentrations of drug. Test compounds were dissolved in deionized water, 50% ethanol, or dimethyl sulfoxide. Serial drug dilutions were made using complete media and 100 μL aliquots were transferred to 96-well clear-bottom black plates. Following addition of 100 μL of asynchronous culture (1% final parasitaemia, 2% final haematocrit), plates were transferred to an airtight chamber, gassed with 5% CO_2_/5% O_2_/90% N_2_ and incubated at 37°C.

For the cytocidal assay, plates were incubated for 6 h followed by centrifugation with an Eppendorf 5804 centrifuge fitted with an A-2-DPW rotor (Hauppauge, NY) at 700 g for 3 min [52]. Drug-containing media was removed and cell pellets washed three times with 200 μL of complete media per wash, using the same centrifuge settings, and re-suspended in the same volume of media. Washed plates were incubated side by side with the cytostatic assay plates at 37°C for 48 h. After 48 h, 50 μL of 10X SYBR Green I dye (diluted using complete media from a 10,000X DMSO stock) was added and plates incubated for an additional 1 h at 37°C to allow DNA intercalation. Fluorescence was measured at 538 nm (485 nm excitation) using a Spectra GeminiEM plate reader (Molecular Devices; Sunnyvale, CA) fitted with a 530 nm long-pass filter. Linear standard curves of measured fluorescence *vs* known parasitaemia were prepared immediately prior to plate analysis. Background controls included fluorescence from un-infected red blood cells. Data was analysed using MS Excel 2007 and IC_50_ and LD_50_ values obtained from sigmoidal curve fits to % growth/survival *vs* drug concentration data using SigmaPlot 11.0. Reported values are the average of three independent assays, with each assay conducted in triplicate (nine determinations total) and reported ± standard error of the mean (S.E.M.), unless otherwise noted.

### Isobologram analysis

The effect of drug combinations was assessed using the modified fixed-ratio isobologram analysis protocol [55] (hereafter referred to as “isobologram analysis”), with some modification. This method detects synergy, additivity, or antagonism between a drug pair. In general, if the drug pair has improved IC_50_ or LD_50_ potency relative to each drug alone, the combination is synergistic, if potency remains unchanged the effect is additive, and if potency is reduced the effect is antagonistic.

Master stock drug solutions were prepared for each drug using complete media, such that the final concentration approximates IC_50_ or LD_50_ following 5–6 twofold dilutions [39,56,57]. Using these master stocks, the following volume-volume (v/v) mixtures of “Drug A” and “Drug B” were prepared: 0:4, 1:3, 1:1, 3:1, and 4:0. These mixtures were then two-fold serially diluted to generate a range of 8 concentrations in each case. Standard cytostatic or cytocidal antiplasmodial assay conditions (see above) were then followed to provide a dose response curve, and IC_50_ or LD_50_ for Drug A and Drug B in each v/v mixture was calculated [33,39,55-61]. Fractional inhibitory concentrations (FICs) were then calculated using Equations 1, 2 and 3 [39,55-57].

(1) $$\mathrm{FI}C_{A}=\frac{IC_{50}\;\mathrm{of}\;\mathrm{Drug}\;A\;\mathrm{in}\;\mathrm{Combination}}{IC_{50}\;\mathrm{of}\;\mathrm{Drug}\;A\;\mathrm{Alone}}$$

(2) $${\mathrm{FIC}}_{B}=\frac{{\mathrm{IC}}_{50}\;\mathrm{of}\;\mathrm{Drug}\;B\;\mathrm{in}\;\mathrm{Combination}}{{\mathrm{IC}}_{50}\;\mathrm{of}\;\mathrm{Drug}\;B\;\mathrm{Alone}}$$

(3) $${\mathrm{FIC}}_{\mathrm{index}}={\mathrm{FIC}}_{A}+{\mathrm{FIC}}_{B}$$

Isobologram curves were constructed by plotting FIC_B_*vs* FIC_A_. A straight diagonal line (FIC_index_ = 1) indicates an additive effect between Drug A and Drug B [39,55-57], concave below the diagonal (FIC_index_ < 1) indicates a synergistic effect and a convex curve above the diagonal (FIC_index_ > 1) indicates antagonism.

## Results

Similar to previous analyses of CQ, QN, QD, MQ and series of CQ and QN analogues [19,20,52], significant differences in cytostatic (IC_50_) and cytocidal (LD_50_) potency are observed for test compounds used in this work (Table 1, see also Additional files 1 and 2). Also similar to previous work [52], differences in drug sensitivity for CQS strain HB3 *vs* CQR strain Dd2 are not of similar magnitude when potency is defined via IC_50_*vs* LD_50_. For example, Dd2 is 2.4-fold resistant to PQ via IC_50_ but approximately three-fold more sensitive via LD_50_ (Table 1). Dd2 shows similar cytostatic sensitivity to TQ compared to HB3, but interestingly is nearly four-fold more sensitive to cytocidal effects of TQ (Table 1). As reported previously, CQR Dd2 is 10-fold cytostatic CQR (“CQR^CS^”) but over 100-fold cytocidal CQR (“CQR^CC^”) [52]. In contrast the related 4-aminoquinoline AQ shows similar potency against both strains, under either cytostatic or cytocidal assay conditions, which is quite rare behaviour for a quinoline anti-malarial drug based on previous examination of many quinoline compounds [19,20,52]. Similar to AQ, MB is quite active relative to quinolines under both cytostatic and cytocidal conditions and shows no major differences between HB3 and Dd2, making it a promising lead compound *vs* CQR *P. falciparum*.

**Table 1:** Anti-plasmodial activity data

	**Experimental IC**_**50**_^**a **^**(nM)**			**Experimental LD**_**50**_^**a **^**(nM)**		
**Drug**	**HB3**	**Dd2**	**R**_**f**_^**b**^	**HB3**	**Dd2**	**R**_**f**_^**b**^
**CQ**	23.8 (0.7)	212.3 (9.0)	9.0	120.0 (10.0)	15300.0 (900.0)	127.5
**AQ**	10.1 (0.6)	26.5 (0.4)	2.6	37.2 (1.2)	52.4 (2.3)	1.4
**PQ**	1990.0 (16.6)	4695.0 (62.1)	2.4	8640.0 (50.0)	2810.0 (10.0)	0.3
**TQ**	2189.9 (18.7)	2092.2 (20.0)	1.0	42700.0 (2042.6)	12100.0 (706.0)	0.3
**MB**	5.3 (0.09)	5.5 (0.07)	1.0	120.2 (9.5)	108.0 (6.6)	0.9

^a^Average of 3 independent measurements, each performed in triplicate (9 determinations total), with S.E.M. reported in parentheses.

^b^Resistance factor (R_f_) = Dd2 value/HB3 value.

**Figure 2:**
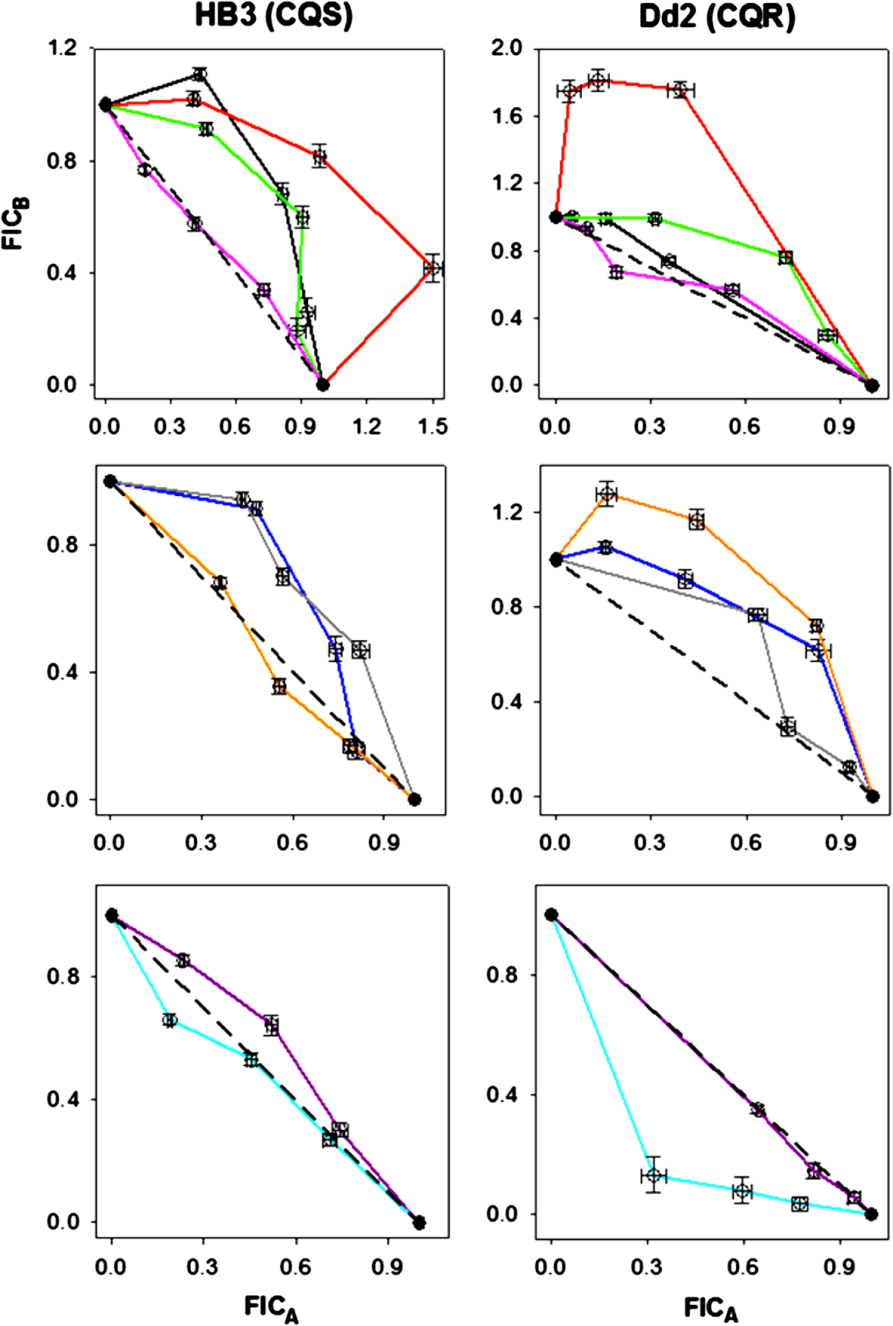
**IC**_**50**_**-based isobologram curves against HB3 (CQS, left column) and Dd2 (CQR, right column) for combinations with CQ (top), AQ (middle), and PQ/TQ (bottom) as “drug A” (x-axes).** CQ-PQ (black), CQ-TQ (red), CQ-MB (green), CQ-AQ (pink), AQ-PQ (dark blue), AQ-TQ (orange), AQ-MB (gray), PQ-MB (purple), and TQ-MB (light blue or cyan). FIC_A_ and FIC_B_ correspond to the fractional inhibitory concentrations (see Methods) of the first and second drugs in each pair listed above, respectively. Error bars represent the standard error of the mean (S.E.M.) for duplicate experiments, each performed in triplicate (6 determinations total).

**Figure 3:**
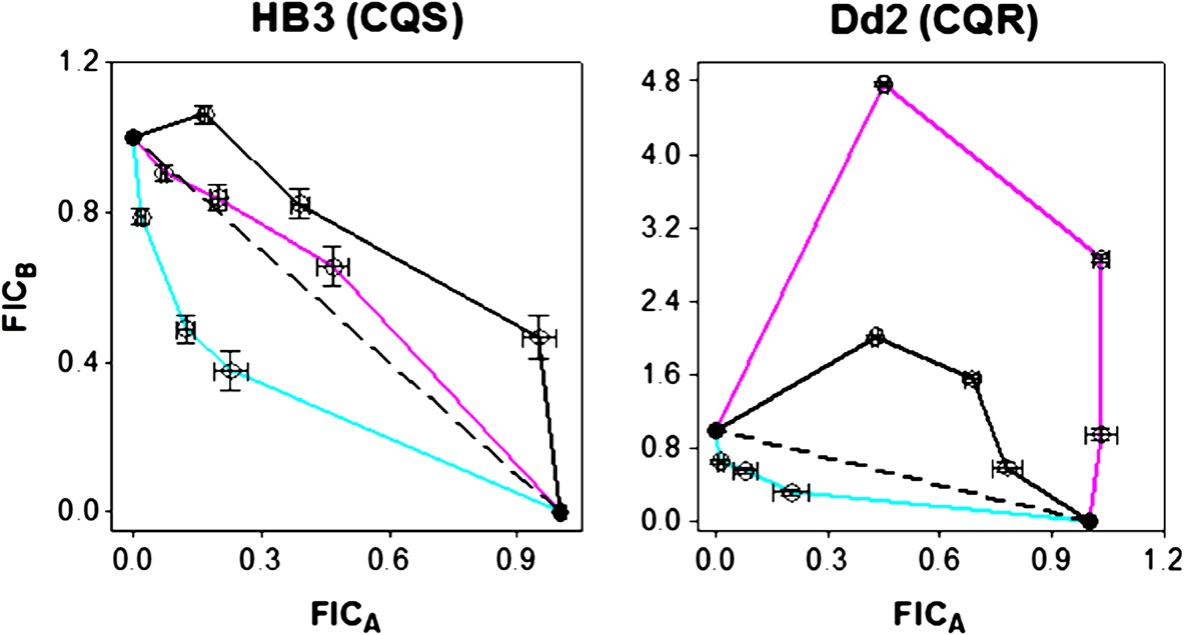
**LD**_**50**_**-based isobologram curves for HB3 (CQS, left) and Dd2 (CQR, right).** CQ-PQ (black), CQ-AQ (pink), and TQ-MB (cyan). FIC_A_ and FIC_B_ correspond to the fractional inhibitory concentrations (see Methods) of the first and second drugs in each pair listed above, respectively. Error bars represent the standard error of the mean (S.E.M.) for duplicate experiments, each performed in triplicate (6 determinations total).

Results of IC_50_- and selected LD_50_-based isobologram analyses of these drugs in various combinations are shown in Figures 2 and 3 (see also Additional files 1, 2 and 3), with antagonism*,* additivity*,* and synergy findings summarized in Table 2. In contrast to Bray *et al.*[33] but in agreement with Akoachere *et al.*[39], the combination of CQ-PQ is antagonistic against HB3 (CQS) (black curve, Figure 2 top left) and additive against Dd2 (CQR) when potency is defined by IC_50_ (black curve, Figure 2 top right). This combination is strongly antagonistic against both strains when potency is defined by LD_50_ (Figure 3, black curves). IC_50_ antagonism is observed with the CQ-TQ combination against both strains (red curves, Figure 2 top) in contrast to synergy seen against the K1 strain (SE Asia, CQR) [33]. AQ combined with PQ is antagonistic against both strains by IC_50_. AQ-TQ is additive against HB3, but antagonistic against Dd2 (orange curves, middle panels Figure 2). CQ-AQ is cytostatically additive for both strains (Figure 2), but exhibits significant antagonism against Dd2 under cytocidal conditions (Figure 3). Overall, the important point is that synergy, antagonism, and additivity seen under cytostatic assay conditions are not necessarily conserved under cytocidal assay conditions. Similar to related conclusions based on multidrug resistance patterns [52] or drug accumulation studies [62], these data further highlight that the mechanism(s) of anti-malarial drug parasite growth inhibition and parasite cell kill are not necessarily the same, and that molecular targets for the cytostatic *vs* cytocidal activities of some important anti-malarial drugs likely differ.

**Table 2:** **Summary of drug interactions**^**a**^

	**IC**_**50**_**-Based**		**LD**_**50**_**-Based**	
**Combination**	**HB3 (CQS)**	**Dd2 (CQR)**	**HB3 (CQS)**	**Dd2 (CQR)**
**CQ-PQ**	Antagonistic	Additive	Antagonistic	Antagonistic
**CQ-TQ**	Antagonistic	Antagonistic	-	-
**CQ-AQ**	Additive	Additive	Additive	Antagonistic
**CQ-MB**	Antagonistic	Antagonistic	-	-
**AQ-PQ**	Antagonistic	Antagonistic	-	-
**AQ-TQ**	Additive	Antagonistic	-	-
**AQ-MB**	Antagonistic	Antagonistic	-	-
**PQ-MB**	Antagonistic	Additive	-	-
**TQ-MB**	Additive	Synergistic	Synergistic	Synergistic

^a^Antagonism is defined by a convex isobologram curve (FIC_index_ > 1), additivity by a isobologram curve along the diagonal (FIC_index_ = 1), and synergy by a concave isobologram curve (FIC_index_ < 1) (Figure 2).

The IC_50_ antagonism observed for CQ-AQ, CQ-MB and AQ-MB combinations is in agreement with previous studies [33,40], except that in the case of AQ-MB, Garavito *et al.* previously measured additive effects for these two drugs against the FcM29 strain (Cameroon, CQR) [40]. IC_50_ antagonism is also observed with PQ-MB against HB3, but additivity is seen against Dd2, in agreement with Garavito *et al.*[40]. Finally, with the exception of IC_50_ additivity against HB3 (Figure 2 bottom left), excellent synergy is seen between TQ and MB under both cytostatic and cytocidal conditions, with synergy being significantly greater in the latter case (Figure 2). Importantly then, some drug combinations may show potent cytostatic and cytocidal synergy for CQR, but not CQS *P. falciparum*. This is an important concept for regions where CQR *P. falciparum* is dominant.

To further explore one contrasting result between these data and previous [33], the CQ-PQ and CQ-TQ combinations were reexamined against the FCB1 strain. This CQR strain from Thailand/S. Africa harbours the same *P. falciparum* chloroquine resistance transporter (PfCRT) amino acid mutations as K1 and has a similar CQ IC_50_[63] (see Additional files 4 and 5). Dd2, however, differs from these two strains in a mutation at position 356, which is I in K1 and FCB1 but T in Dd2. Mutations in the *P. falciparum* multi-drug resistance protein (PfMDR1) are identical among these three strains [64,65] (see Additional file 6). Results obtained with FCB1 are similar to those obtained with HB3 and Dd2, in that combination of CQ with either PQ or TQ is antagonistic (Table 2, see also Additional file 5).

## Discussion

This study reports the first systematic analysis of quinoline anti-malarial drug combinations quantified for both cytostatic and cytocidal potency. Recent studies suggest that cytostatic and cytocidal mechanisms of action for some anti-malarial drugs differ [19,20,52,62], and the present data further support this concept. Comparative investigation of drug combinations under both IC_50_ and LD_50_ conditions will enhance further development of effective combination therapies and also increase understanding of antimalarial drug pharmacology and resistance. IC_50_ and LD_50_ data for the individual drugs reinforces this concept (Table 1). Strain Dd2 is 10-fold resistant to CQ (relative to strain HB3) when IC_50_ is ratio’d, but over 100-fold resistant when LD_50_ values are ratio’d. On the other hand, the closely-related 4-aminoquinoline AQ has similar potency for both strains under both cytostatic and cytocidal conditions, a characteristic also found for ART (A. Gorka, P. Roepe, unpublished). MB, while having nearly identical activity for the CQS and CQR strains (behavior similar to AQ), is about 20-fold less potent by LD_50_ relative to IC_50_ (unlike AQ). PQ, an 8-aminoquinoline known to be a poor blood schizonticide [17,18], is nonetheless active compared to CQ when potency is defined by LD_50_ for CQR strain Dd2 (15.3 μM CQ LD_50_*vs* 2.8 μM PQ LD_50_, Table 1). In fact, Dd2 is about three-fold hypersensitive to PQ relative to HB3 when LD_50_ are compared. Results for drug combinations show significant differences in synergy, additivity, and antagonism (Table 2), as well as in the shapes of their isobologram curves (Figure 2). In general, cytocidal antagonism is significantly more pronounced relative to examples of cytostatic antagonism. Overall, these data offer additional explanations for why *in vitro* IC_50_-based assessment of mono- or combination therapy may not always predict results in the clinic, where both cytostatic and cytocidal potencies are critical.

The CQ-PQ combination results in cytostatic antagonism (CQS) and additivity (CQR), which disagrees with data [33] for the CQR strain K1, where potent synergy for CQ-PQ and CQ-TQ combinations was found. In growth inhibition assays, as suggested [33], the combination effects may be PfCRT-dependent and rely on the presence of the PQ/TQ basic side chain, as well as co-administration of the drug pair [33]. As suggested [33], due to their structural similarity to CQ but increased lipophilicity, PQ and TQ may bind with higher affinity to the mutated pore of PfCRT and block passage of CQ [33]. However, compared to strain Dd2, the K1 strain (CQR, S.E. Asia) differs in a PfCRT mutation at position 356 (see Additional file 4) [63]. Although strain K1 was not available for this study, CQ-PQ and CQ-TQ combinations were re-tested against the FCB1 strain (CQR, Thailand/S. Africa), which bears an identical set of PfCRT [63] and PfMDR1 [64,65] mutations relative to K1. These combinations again exhibited antagonism (see Additional file 5), indicating altered CQ transport by PfCRT may not be the only factor governing combination effects.

The closely-related 4-aminoquinoline AQ also showed antagonism with PQ and TQ across all conditions, with the exception of AQ-TQ against Dd2, which was additive. Therefore, similar to the CQ combinations discussed above, differences between cytostatic and cytocidal response, as well as important differences between the CQS and CQR strains are observed when AQ is a partner drug. Further exploration of these concepts with other CQS and CQR strains is vital. Combination of CQ with AQ was purely additive against both strains by IC_50_ and against the CQS strain by LD_50_ (Figures 2 and 3, Table 2). AQ is expected to have a similar overall mechanism of action to that of CQ under cytostatic conditions (e.g. inhibition of Hz formation), so additive action of these two drugs in combination is expected for IC_50_. Surprisingly though, CQ-AQ exhibits significant antagonism against the CQR Dd2 strain by LD_50_. In light of recent evidence [19,20,52,62], this observation again points to the general hypothesis that drug targets and the manner in which the parasite facilitates resistance to those drugs are different under cytocidal *vs* cytostatic conditions. In investigating this further, future work will test for synergy, additivity, and antagonism for these drug pairs against a wider range of strains harbouring different CQR-associated PfCRT alleles.

CQ-MB was cytostatically antagonistic against both strains, in agreement with Akoachere *et al.*, who observed antagonism against 3D7 (CQS) and K1 [39], but in contrast to Garavito *et al.*, who observed additivity against the FcM29 strain (CQR, Cameroon) [40]. Antagonism between CQ and MB is surprising given the high blood schizonticidal potency of MB, its potent inhibition of Hz formation *in vitro*[41,42], and inhibition of parasite glutathione reductase [46-48,66,67], as well as its ability to accumulate in the DV, similar to CQ [42]. However, such antagonism has also been reported in the field, with failure of CQ and MB observed in treatment of uncomplicated malaria in young children in Burkina Faso [49,50]. AQ was also antagonistic with MB, in agreement with Garavito *et al.*[40]. IC_50_ antagonism in such cases might indicate distinctly different modes of haemozoin precursor binding [68].

## Conclusions

Using a rigorous isobologram approach, cytocidal and cytostatic anti-plasmodial potency have been determined for various combinations of quinoline drugs and MB, for both a CQS and CQR strain of *P. falciparum*. The results highlight differences in drug activity and resistance under cytostatic *vs* cytocidal conditions. They also show that antagonism or synergy found for a drug pair via IC_50_ data (cytostatic activity) does not necessarily predict the same behavior when potency for the pair is quantified via LD_50_ data (cytocidal activity). Furthermore, the data help prioritize combinations that warrant further exploration against CQR malaria (e.g. TQ-MB), *vs* those with limited applicability (e.g. CQ-PQ). Important next steps are to examine the correlation between anti-malarial drug combination effects and Hz inhibition, as well as to widen this cytostatic *vs* cytocidal profiling to other drugs, active drug metabolites, and many geographic isolates of *P. falciparum*.

## Abbreviations

ACT: Artemisinin combination therapy; AQ: Amodiaquine; ART: Artemisinin; CQ: Chloroquine; CQR: Chloroquine resistant; CQRCC: Cytocidal chloroquine resistance; CQS: Chloroquine sensitive; CQSCC: Cytostatic chloroquine resistance; DV: Digestive vacuole; FIC: Fractional inhibitory concentration; G6PD: Glucose-6-phosphate dehydrogenase; GR: Glutathione reductase; Hz: Haemozoin; MB: Methylene blue; MQ: Mefloquine; QN: Quinine; PfCRT: *Plasmodium falciparum* chloroquine resistance transporter; PfMDR1: *Plasmodium falciparum* multi-drug-resistance protein; PQ: Primaquine; TQ: Tafenoquine; vs: *Versus*

## Competing interests

The authors declare they have no competing interests.

## Authors’ contributions

APG carried out antiplasmodial and isobologram assays and data analysis, assisted in interpretation of the results, and drafted the manuscript. LMJ maintained *P. falciparum* cultures, participated in anti-plasmodial and isobologram assays and data analysis, and assisted manuscript preparation. PDR conceived of the study, interpreted results, and drafted the manuscript. All authors have read and approved the final manuscript.

## Supplementary Material

Additional file 1 **IC**_**50 **_**data for all drug combinations tested against HB3 and Dd2.** Click here for file

Additional file 2 **LD**_**50 **_**data for all drug combinations tested against HB3 and Dd2.** Click here for file

Additional file 3 **Results of IC**_**50**_**-based drug combination analyses.** All FIC data is shown with relevant statistics. Click here for file

Additional file 4 **PfCRT amino acid substitutions associated with CQS and CQR PfCRT isoforms in *Plsmodium falciparum.***
Click here for file

Additional file 5 **IC**_**50 **_**data for all drug combinations tested against FCB1.** Click here for file

Additional file 6 **PfMDR1 amino acid substitutions associated with CQS and CQR PfMDR1 isoforms in *Plasmodium falciparum.***
Click here for file
