# Supplementary material for: Cytostatic versus cytocidal profiling of quinoline drug combinations via modified fixed-ratio isobologram analysis
Source: Malar J. 2013 Sep 18;12:332. doi: 10.1186/1475-2875-12-332 (PMC3874740; doi:10.1186/1475-2875-12-332)
Supplement: Additional file 1 — IC 50 data for all drug combinations tested against HB3 and Dd2. [file 1475-2875-12-332-S1.doc]

**Additional File 1.** IC_50_ data for all drug combinations against HB3 and Dd2.

|  |  | **IC_50_ (nM)** | | | | | | | |
| --- | --- | --- | --- | --- | --- | --- | --- | --- | --- |
|  |  | **Trial I^c^** | | **Trial II^c^** | | **Average** | | | |
| **Combination^a^** | **Drug^b^** | **HB3** | **Dd2** | **HB3** | **Dd2** | **HB3** | **S.E.M.^d^** | **Dd2** | **S.E.M.^d^** |
| **0:4 CQ-PQ** | **CQ** | - | - | - | - | - | - | - | - |
|  | **PQ** | 5699.6 | 2655.2 | 5991.0 | 2695.8 | 5845.3 | 145.7 | 2675.5 | 20.3 |
| **1:3 CQ-PQ** | **CQ** | 15.7 | 32.9 | 15.7 | 32.0 | 15.7 | 0.0 | 32.5 | 0.4 |
|  | **PQ** | 6462.1 | 2712.2 | 6481.2 | 2639.5 | 6471.7 | 9.5 | 2675.9 | 36.3 |
| **1:1 CQ-PQ** | **CQ** | 29.9 | 101.6 | 28.6 | 90.6 | 29.3 | 0.6 | 96.1 | 5.5 |
|  | **PQ** | 4037.6 | 2792.9 | 3936.6 | 2491.4 | 3987.1 | 50.5 | 2642.2 | 150.8 |
| **3:1 CQ-PQ** | **CQ** | 32.3 | 213.7 | 34.3 | 216.0 | 33.3 | 1.0 | 214.9 | 1.2 |
|  | **PQ** | 1481.6 | 1959.1 | 1574.3 | 1979.6 | 1528.0 | 46.4 | 1969.4 | 10.3 |
| **4:0 CQ-PQ** | **CQ** | 35.9 | 607.1 | 36.2 | 593.5 | 36.1 | 0.2 | 600.3 | 6.8 |
|  | **PQ** | - | - | - | - | - | - | - | - |
| **0:4 CQ-TQ** | **CQ** | - | - | - | - | - | - | - | - |
|  | **TQ** | 5007.5 | 915.5 | 4998.7 | 1526.3 | 5003.1 | 4.4 | 1220.9 | 305.4 |
| **1:3 CQ-TQ** | **CQ** | 13.6 | 30.3 | 13.6 | 26.6 | 13.6 | 0.0 | 28.5 | 1.9 |
|  | **TQ** | 5109.0 | 2272.7 | 5109.4 | 1998.7 | 5109.2 | 0.2 | 2135.7 | 137.0 |
| **1:1 CQ-TQ** | **CQ** | 33.1 | 89.3 | 32.2 | 87.6 | 32.7 | 0.4 | 88.5 | 0.9 |
|  | **TQ** | 4137.4 | 2231.4 | 4029.9 | 2190.7 | 4083.7 | 53.7 | 2211.1 | 20.4 |
| **3:1 CQ-TQ** | **CQ** | 51.5 | 263.6 | 48.2 | 251.1 | 49.9 | 1.7 | 257.4 | 6.3 |
|  | **TQ** | 2146.2 | 2196.5 | 2007.0 | 2092.5 | 2076.6 | 69.6 | 2144.5 | 52.0 |
| **4:0 CQ-TQ** | **CQ** | 33.1 | 622.3 | 33.3 | 677.5 | 33.2 | 0.1 | 649.9 | 27.6 |
|  | **TQ** | - | - | - | - | - | - | - | - |
| **0:4 CQ-MB** | **CQ** | - | - | - | - | - | - | - | - |
|  | **MB** | 13.9 | 33.9 | 11.3 | 33.7 | 12.6 | 1.3 | 33.8 | 0.1 |
| **1:3 CQ-MB** | **CQ** | 15.4 | 229.1 | 15.4 | 218.3 | 15.4 | 0.0 | 223.7 | 5.4 |
|  | **MB** | 11.5 | 34.4 | 11.5 | 32.7 | 11.5 | 0.0 | 33.6 | 0.8 |
| **1:1 CQ-MB** | **CQ** | 30.4 | 512.7 | 30.0 | 513.9 | 30.2 | 0.2 | 513.3 | 0.6 |
|  | **MB** | 7.6 | 25.6 | 7.5 | 25.7 | 7.6 | 0.0 | 25.7 | 0.0 |
| **3:1 CQ-MB** | **CQ** | 29.3 | 728.0 | 29.4 | 484.2 | 29.4 | 0.0 | 606.1 | 121.9 |
|  | **MB** | 2.4 | 12.1 | 2.5 | 8.1 | 2.5 | 0.1 | 10.1 | 2.0 |
| **4:0 CQ-MB** | **CQ** | 33.4 | 725.7 | 33.3 | 684.4 | 33.4 | 0.1 | 705.1 | 20.7 |
|  | **MB** | - | - | - | - | - | - | - | - |
| **0:4 CQ-AQ** | **CQ** | - | - | - | - | - | - | - | - |
|  | **AQ** | 19.9 | 33.4 | 18.4 | 35.8 | 19.2 | 0.8 | 34.6 | 1.2 |
| **1:3 CQ-AQ** | **CQ** | 6.6 | 74.7 | 6.5 | 68.5 | 6.6 | 0.0 | 71.6 | 3.1 |
|  | **AQ** | 14.8 | 33.6 | 14.6 | 30.8 | 14.7 | 0.1 | 32.2 | 1.4 |
| **1:1 CQ-AQ** | **CQ** | 14.6 | 136.5 | 14.8 | 137.7 | 14.7 | 0.1 | 137.1 | 0.6 |
|  | **AQ** | 10.9 | 26.0 | 11.1 | 20.6 | 11.0 | 0.1 | 23.3 | 2.7 |
| **3:1 CQ-AQ** | **CQ** | 25.9 | 372.4 | 26.0 | 416.9 | 26.0 | 0.1 | 394.7 | 22.3 |
|  | **AQ** | 6.5 | 18.6 | 6.5 | 20.8 | 6.5 | 0.0 | 19.7 | 1.1 |
| **4:0 CQ-AQ** | **CQ** | 35.6 | 705.3 | 35.9 | 705.9 | 35.8 | 0.1 | 705.6 | 0.3 |
|  | **AQ** | - | - | - | - | - | - | - | - |
| **0:4 AQ-PQ** | **AQ** | - | - | - | - | - | - | - | - |
|  | **PQ** | 6157.2 | 2687.9 | 5535.6 | 2696.7 | 5846.4 | 310.8 | 2692.3 | 4.4 |
| **1:3 AQ-PQ** | **AQ** | 9.4 | 5.2 | 10.0 | 5.1 | 9.7 | 0.3 | 5.2 | 0.1 |
|  | **PQ** | 5163.9 | 2850.8 | 5514.9 | 2817.4 | 5339.4 | 175.5 | 2834.1 | 16.7 |
| **1:1 AQ-PQ** | **AQ** | 15.1 | 13.6 | 15.0 | 13.2 | 15.1 | 0.0 | 13.4 | 0.2 |
|  | **PQ** | 2767.5 | 2501.9 | 2734.3 | 2428.9 | 2750.9 | 16.6 | 2465.4 | 36.5 |
| **3:1 AQ-PQ** | **AQ** | 16.5 | 27.1 | 16.4 | 27.1 | 16.5 | 0.1 | 27.1 | 0.0 |
|  | **PQ** | 884.9 | 1657.7 | 886.5 | 1656.4 | 885.7 | 0.8 | 1657.1 | 0.6 |
| **4:0 AQ-PQ** | **AQ** | 21.2 | 32.3 | 19.4 | 33.2 | 20.3 | 0.9 | 32.8 | 0.5 |
|  | **PQ** | - | - | - | - | - | - | - | - |
| **0:4 AQ-TQ** | **AQ** | - | - | - | - | - | - | - | - |
|  | **TQ** | 5372.7 | 1864.3 | 5178.7 | 1866.8 | 5275.7 | 97.0 | 1865.6 | 1.3 |
| **1:3 AQ-TQ** | **AQ** | 7.7 | 4.8 | 6.9 | 4.7 | 7.3 | 0.4 | 4.8 | 0.0 |
|  | **TQ** | 3827.6 | 2396.1 | 3346.2 | 2370.3 | 3586.9 | 240.7 | 2383.2 | 12.9 |
| **1:1 AQ-TQ** | **AQ** | 11.5 | 13.1 | 11.0 | 13.1 | 11.3 | 0.3 | 13.1 | 0.0 |
|  | **TQ** | 1910.9 | 2176.3 | 1827.1 | 2185.4 | 1869.0 | 41.9 | 2180.9 | 4.5 |
| **3:1 AQ-TQ** | **AQ** | 16.0 | 24.4 | 15.9 | 23.9 | 16.0 | 0.0 | 24.2 | 0.3 |
|  | **TQ** | 885.5 | 1356.2 | 885.6 | 1328.0 | 885.6 | 0.1 | 1342.1 | 14.1 |
| **4:0 AQ-TQ** | **AQ** | 20.4 | 28.8 | 20.1 | 30.1 | 20.3 | 0.1 | 29.5 | 0.7 |
|  | **TQ** | - | - | - | - | - | - | - | - |
| **0:4 AQ-MB** | **AQ** | - | - | - | - | - | - | - | - |
|  | **MB** | 9.5 | 25.5 | 9.7 | 26.2 | 9.6 | 0.1 | 25.9 | 0.4 |
| **1:3 AQ-MB** | **AQ** | 9.0 | 19.9 | 9.1 | 19.7 | 9.1 | 0.0 | 19.8 | 0.1 |
|  | **MB** | 9.0 | 19.9 | 9.1 | 19.7 | 9.1 | 0.0 | 19.8 | 0.1 |
| **1:1 AQ-MB** | **AQ** | 11.6 | 23.3 | 12.0 | 22.2 | 11.8 | 0.2 | 22.8 | 0.6 |
|  | **MB** | 3.9 | 7.8 | 4.0 | 7.4 | 4.0 | 0.1 | 7.6 | 0.2 |
| **3:1 AQ-MB** | **AQ** | 17.5 | 27.8 | 16.7 | 29.8 | 17.1 | 0.4 | 28.8 | 1.0 |
|  | **MB** | 1.9 | 3.1 | 7.1 | 3.3 | 4.5 | 2.6 | 3.2 | 0.1 |
| **4:0 AQ-MB** | **AQ** | 21.4 | 31.4 | 20.2 | 30.8 | 20.8 | 0.6 | 31.1 | 0.3 |
|  | **MB** | - | - | - | - | - | - | - | - |
| **0:4 PQ-MB** | **PQ** | - | - | - | - | - | - | - | - |
|  | **MB** | 8.4 | 23.2 | 8.1 | 22.2 | 8.3 | 0.2 | 22.7 | 0.5 |
| **1:3 PQ-MB** | **PQ** | 1329.0 | 1423.9 | 1260.2 | 1490.4 | 1294.6 | 34.4 | 1457.2 | 33.3 |
|  | **MB** | 7.2 | 7.8 | 6.9 | 8.1 | 7.1 | 0.2 | 8.0 | 0.2 |
| **1:1 PQ-MB** | **PQ** | 2934.9 | 1883.3 | 2900.3 | 1810.0 | 2917.6 | 17.3 | 1846.7 | 36.7 |
|  | **MB** | 5.3 | 3.4 | 5.3 | 3.3 | 5.3 | 0.0 | 3.4 | 0.1 |
| **3:1 PQ-MB** | **PQ** | 4308.4 | 2124.6 | 4016.0 | 2132.2 | 4162.2 | 146.2 | 2128.4 | 3.8 |
|  | **MB** | 2.6 | 1.3 | 2.4 | 1.3 | 2.5 | 0.1 | 1.3 | 0.0 |
| **4:0 PQ-MB** | **PQ** | 5397.1 | 2254.8 | 5776.2 | 2255.0 | 5586.7 | 189.6 | 2254.9 | 0.1 |
|  | **MB** | - | - | - | - | - | - | - | - |
| **0:4 TQ-MB** | **TQ** | - | - | - | - | - | - | - | - |
|  | **MB** | 9.0 | 27.9 | 8.7 | 28.3 | 8.9 | 0.2 | 28.1 | 0.2 |
| **1:3 TQ-MB** | **TQ** | 985.1 | 610.5 | 974.7 | 614.9 | 979.9 | 5.2 | 612.7 | 2.2 |
|  | **MB** | 5.9 | 3.7 | 5.8 | 3.7 | 5.9 | 0.1 | 3.7 | 0.0 |
| **1:1 TQ-MB** | **TQ** | 2335.6 | 1173.6 | 2349.4 | 1119.1 | 2342.5 | 6.9 | 1146.4 | 27.3 |
|  | **MB** | 4.7 | 2.3 | 4.7 | 2.2 | 4.7 | 0.0 | 2.3 | 0.0 |
| **3:1 TQ-MB** | **TQ** | 3716.4 | 1518.4 | 3606.1 | 1459.6 | 3661.3 | 55.2 | 1489.0 | 29.4 |
|  | **MB** | 2.4 | 1.0 | 2.4 | 1.0 | 2.4 | 0.0 | 1.0 | 0.0 |
| **4:0 TQ-MB** | **TQ** | 5209.3 | 1859.2 | 5068.2 | 1976.2 | 5138.8 | 70.6 | 1917.7 | 58.5 |
|  | **MB** | - | - | - | - | - | - | - | - |

^a^Volume-volume (v/v) mixtures (see Methods).

^b^CQ – chloroquine, AQ – amodiaquine, PQ – primaquine, TQ – tafenoquine, MB – methylene blue.

^c^Result of 3 replicates.

^d^S.E.M. – standard error of the mean.
