# Supplementary material for: Cytostatic versus cytocidal profiling of quinoline drug combinations via modified fixed-ratio isobologram analysis
Source: Malar J. 2013 Sep 18;12:332. doi: 10.1186/1475-2875-12-332 (PMC3874740; doi:10.1186/1475-2875-12-332)
Supplement: Additional file 2 — LD 50 data for all drug combinations tested against HB3 and Dd2. [file 1475-2875-12-332-S2.doc]

**Additional File 2.** LD_50_ data for all drug combinations tested against HB3 and Dd2.

^a^Volume-volume (v/v) mixtures (see Methods).

|  |  |  | | | |
| --- | --- | --- | --- | --- | --- |
|  |  | **LD_50_ (nM)^c^** | | | |
| **Combination^a^** | **Drug^b^** | **HB3** | **S.E.M.^d^** | **Dd2** | **S.E.M.^d^** |
| **0:4 CQ-PQ** | **CQ** | - | - | - | - |
|  | **PQ** | 10359.0 | 380.1 | 4656.8 | 77.2 |
| **1:3 CQ-PQ** | **CQ** | 97.7 | 10.2 | 15078.2 | 444.5 |
|  | **PQ** | 10993.6 | 200.9 | 9375.0 | 95.6 |
| **1:1 CQ-PQ** | **CQ** | 227.1 | 15.8 | 24200.6 | 503.5 |
|  | **PQ** | 8515.2 | 112.4 | 7260.2 | 146.0 |
| **3:1 CQ-PQ** | **CQ** | 553.0 | 41.3 | 27498.1 | 601.4 |
|  | **PQ** | 6912.2 | 100.1 | 2749.8 | 569.6 |
| **4:0 CQ-PQ** | **CQ** | 581.3 | 52.2 | 35270.1 | 640.5 |
|  | **PQ** | - | - | - | - |
| **0:4 CQ-AQ** | **CQ** | - | - | - | - |
|  | **AQ** | 41.5 | 6.3 | 42.0 | 4.1 |
| **1:3 CQ-AQ** | **CQ** | 33.4 | 5.5 | 17486.1 | 231.9 |
|  | **AQ** | 37.6 | 3.6 | 200.0 | 33.0 |
| **1:1 CQ-AQ** | **CQ** | 92.7 | 11.6 | 40115.6 | 700.7 |
|  | **AQ** | 34.8 | 1.4 | 120.3 | 9.8 |
| **3:1 CQ-AQ** | **CQ** | 217.4 | 12.1 | 40091.5 | 722.5 |
|  | **AQ** | 27.2 | 2.0 | 40.1 | 6.4 |
| **4:0 CQ-AQ** | **CQ** | 463.6 | 20.6 | 38845.3 | 662.8 |
|  | **AQ** | - | - | - | - |
| **0:4 TQ-MB** | **TQ** | - | - | - | - |
|  | **MB** | 109.6 | 13.9 | 80.5 | 7.8 |
| **1:3 TQ-MB** | **TQ** | 1483.0 | 112.0 | 922.9 | 84.7 |
|  | **MB** | 86.3 | 9.7 | 52.8 | 5.8 |
| **1:1 TQ-MB** | **TQ** | 9294.8 | 203.0 | 5685.9 | 176.4 |
|  | **MB** | 53.6 | 1.9 | 44.9 | 2.3 |
| **3:1 TQ-MB** | **TQ** | 17319.7 | 391.2 | 14519.9 | 296.9 |
|  | **MB** | 41.3 | 6.1 | 25.7 | 0.9 |
| **4:0 TQ-MB** | **TQ** | 75654.2 | 1128.5 | 71726.6 | 1820.3 |
|  | **MB** | - | - | - | - |

^b^CQ – chloroquine, AQ – amodiaquine, PQ – primaquine, TQ – tafenoquine, MB – methylene blue.

^c^Result of duplicate experiments, each performed in duplicate (4 determinations total.

^d^S.E.M. – standard error of the mean
