# Supplementary material for: Cytostatic versus cytocidal profiling of quinoline drug combinations via modified fixed-ratio isobologram analysis
Source: Malar J. 2013 Sep 18;12:332. doi: 10.1186/1475-2875-12-332 (PMC3874740; doi:10.1186/1475-2875-12-332)
Supplement: Additional file 3 — Results of IC 50 -based drug combination analyses. All FIC data is shown with relevant statistics. [file 1475-2875-12-332-S3.doc]

**Additional File 3.** Results of IC_50_-based drug combination analyses.

|  |  | **IC_50_-Based^b^** | | | | | | **LD_50_-Based^c^** | | | | | |
| --- | --- | --- | --- | --- | --- | --- | --- | --- | --- | --- | --- | --- | --- |
|  |  | **FIC_A_** | | **FIC_B_** | |  |  | **FIC_A_** | | **FIC_B_** | |  |  |
| **Drug**  **(A-B)** | **Combination^a^** | **HB3** | **Dd2** | **HB3** | **Dd2** | **FIC_Index_**  **HB3** | **FIC_Index_**  **Dd2** | **HB3** | **Dd2** | **HB3** | **Dd2** | **FIC_Index_**  **HB3** | **FIC_Index_**  **Dd2** |
| **CQ-PQ** | 1:3  1:1  3:1 | 0.436  (0.009)  0.811  (0.021)  0.924  (0.040) | 0.054  (0.038)  0.160  (0.031)  0.358  (0.041) | 1.107  (0.023)  0.682  (0.040)  0.261  (0.048) | 1.000  (0.064)  0.988  (0.061)  0.736  (0.050) | 1.5  1.5  1.2 | 1.1  1.1  1.1 | 0.168 (0.010)  0.391 (0.021)  0.951 (0.040) | 0.428 (0.009)  0.686 (0.020)  0.780 (0.038) | 1.061 (0.023)  0.822 (0.040)  0.667 (0.058) | 2.013 (0.022)  1.560 (0.039)  0.590 (0.057) | 1.2  1.2  1.6 | 2.4  2.2  1.4 |
| **CQ-TQ** | 1:3  1:1  3:1 | 0.410  (0.010)  0.983  (0.022)  1.502  (0.042) | 0.044  (0.033)  0.136  (0.022)  0.396  (0.010) | 1.021  (0.024)  0.816  (0.042)  0.415  (0.051) | 1.749  (0.052)  1.811  (0.043)  1.756  (0.025) | 1.4  1.8  1.9 | 1.8  1.9  2.2 | - | - | - | - | - | - |
| **CQ-AQ** | 1:3  1:1  3:1 | 0.183  (0.009)  0.411  (0.020)  0.726  (0.028) | 0.101  (0.009)  0.194  (0.021)  0.559  (0.030) | 0.768  (0.023)  0.574  (0.038)  0.339  (0.027) | 0.931  (0.023)  0.673  (0.050)  0.569  (0.028) | 1.0  1.0  1.1 | 1.0  0.9  1.1 | 0.072 (0.009)  0.200 (0.019)  0.469 (0.036) | 0.450 (0.010)  1.033 (0.021)  1.032 (0.040) | 0.906 (0.021)  0.839 (0.036)  0.655 (0.053) | 4.762 (0.024)  2.864 (0.041)  0.955 (0.059) | 1.0  1.0  1.1 | 5.5  3.9  2.0 |
| **CQ-MB** | 1:3  1:1  3:1 | 0.462  (0.009)  0.906  (0.020)  0.880  (0.038) | 0.317  (0.009)  0.728  (0.021)  0.860  (0.040) | 0.913  (0.023)  0.599  (0.039)  0.194  (0.047) | 0.993  (0.023)  0.759  (0.040)  0.299  (0.048) | 1.4  1.5  1.1 | 1.3  1.5  1.2 | - | - | - | - | - | - |
| **AQ-PQ** | 1:3  1:1  3:1 | 0.478  (0.008)  0.741  (0.019)  0.810  (0.027) | 0.157  (0.028)  0.409  (0.020)  0.827  (0.009) | 0.913  (0.022)  0.471  (0.027)  0.151  (0.026) | 1.053  (0.027)  0.916  (0.038)  0.615  (0.022) | 1.4  1.2  1.0 | 1.2  1.3  1.4 | - | - | - | - | - | - |
| **AQ-TQ** | 1:3  1:1  3:1 | 0.360  (0.008)  0.556  (0.018)  0.788  (0.025) | 0.161  (0.040)  0.445  (0.028)  0.820  (0.026) | 0.680  (0.020)  0.354  (0.034)  0.168  (0.024) | 1.277  (0.060)  1.169  (0.050)  0.719  (0.024) | 1.0  0.9  1.0 | 1.4  1.6  1.5 | - | - | - | - | - | - |
| **AQ-MB** | 1:3  1:1  3:1 | 0.435  (0.007)  0.567  (0.016)  0.822  (0.023) | 0.637  (0.007)  0.732  (0.016)  0.926  (0.022) | 0.943  (0.018)  0.411  (0.021)  0.469  (0.021) | 0.766  (0.018)  0.294  (0.030)  0.124  (0.021) | 1.4  1.0  1.3 | 1.4  1.0  1.0 | - | - | - | - | - | - |
| **PQ-MB** | 1:3  1:1  3:1 | 0.232  (0.007)  0.522  (0.015)  0.745  (0.022) | 0.646  (0.007)  0.819  (0.015)  0.944  (0.021) | 0.855  (0.017)  0.642  (0.025)  0.303  (0.020) | 0.350  (0.017)  0.148  (0.029)  0.057  (0.020) | 1.1  1.2  1.0 | 1.0  1.0  1.0 | - | - | - | - | - | - |
| **TQ-MB** | 1:3  1:1  3:1 | 0.191  (0.006)  0.456  (0.015)  0.712  (0.021) | 0.319  (0.006)  0.598  (0.014)  0.776  (0.019) | 0.661  (0.016)  0.531  (0.023)  0.271  (0.019) | 0.132  (0.015)  0.080  (0.027)  0.036  (0.008) | 0.9  1.0  1.0 | 0.5  0.7  0.8 | 0.020 (0.009)  0.123 (0.020)  0.229 (0.037) | 0.013 (0.091)  0.079 (0.030)  0.202 (0.048) | 0.787 (0.022)  0.489 (0.038)  0.377 (0.056) | 0.656 (0.022)  0.558 (0.028)  0.319 (0.036) | 0.8  0.6  0.6 | 0.7  0.6  0.5 |

^a^Volume-Volume (v/v) mixtures (see Methods).

^b^Result of duplicate measurements, each performed in triplicate (6 determinations total), with S.E.M. shown in parentheses.

^c^Result of duplicate experiments, each performed in duplicate (4 determinations total), with S.E.M. shown in parantheses
