# Supplementary material for: Cytostatic versus cytocidal profiling of quinoline drug combinations via modified fixed-ratio isobologram analysis
Source: Malar J. 2013 Sep 18;12:332. doi: 10.1186/1475-2875-12-332 (PMC3874740; doi:10.1186/1475-2875-12-332)
Supplement: Additional file 4 — PfCRT amino acid substitutions associated with CQS and CQR PfCRT isoforms in Plsmodium falciparum. [file 1475-2875-12-332-S4.doc]

**Additional File 4.** PfCRT amino acid substitutions associated with CQS and CQR PfCRT isoforms in *Plasmodium falciparum*.a

|  | | | **PfCRT Amino Acid Positions** | | | | | | | | | | | | | | | | | | |
| --- | --- | --- | --- | --- | --- | --- | --- | --- | --- | --- | --- | --- | --- | --- | --- | --- | --- | --- | --- | --- | --- |
| **Clone/**  **Isolate** | **Origin** | **Classification** | **72** | **74** | **75** | **76** | **97** | **144** | **148** | **152** | **160** | **163** | **194** | **220** | **271** | **275** | **326** | **333** | **352** | **356** | **371** |
| **HB3** | Honduras | CQS | C | M | N | K | H | A | L | T | L | S | I | A | Q | P | N | T | Q | I | R |
| **K1** | S.E. Asia | CQR | C | I | E | T | H | A | L | T | L | S | I | S | E | P | S | T | Q | I | I |
| **FCB** | Thailand/  S. Africa | CQR | C | I | E | T | H | A | L | T | L | S | I | S | E | P | S | T | Q | I | I |
| **Dd2** | Indochina | CQR | C | I | E | T | H | A | L | T | L | S | I | S | E | P | S | T | Q | T | I |

^a^Fidock, D. A.; Nomura, T.; Talley, A. K.; Cooper, R. A.; Dzekunov, S. M.; Ferdig, M. T.; Ursos, L. M.; Sidhu, A. B.; Naudé, B.; Deitsch, K. W.; Su, X. Z.; Wootton, J. C.; Roepe, P. D.; Wellems, T. E. *Mol. Cell* **2000**, *6*, 861-871.
