# Supplementary material for: Cytostatic versus cytocidal profiling of quinoline drug combinations via modified fixed-ratio isobologram analysis
Source: Malar J. 2013 Sep 18;12:332. doi: 10.1186/1475-2875-12-332 (PMC3874740; doi:10.1186/1475-2875-12-332)
Supplement: Additional file 5 — IC 50 data for all drug combinations tested against FCB1. [file 1475-2875-12-332-S5.doc]

**Additional File 5.** IC_50_ data for all drug combinations tested against FCB.

|  |  |  | | | |
| --- | --- | --- | --- | --- | --- |
| **Combination^a^** | **Drug^b^** | **IC_50_ (nM)^c^** | **S.E.M.^d^** | **FIC^e^** | **FIC_index_^f^** |
| **0:4 CQ-PQ** | **CQ** | - | - | - | - |
|  | **PQ** | 2608.9 | 139.2 | - |  |
| **1:3 CQ-PQ** | **CQ** | 24.05 | 5.7 | 0.034  (0.001) | 1.1 |
|  | **PQ** | 2790.8 | 64.0 | 1.071  (0.023) |  |
| **1:1 CQ-PQ** | **CQ** | 88.0 | 16.7 | 0.118  (0.002) | 1.4 |
|  | **PQ** | 3213.7 | 296.8 | 1.235  (0.040) |  |
| **3:1 CQ-PQ** | **CQ** | 321.9 | 35.1 | 0.263  (0.009) | 4.0 |
|  | **PQ** | 9585.1 | 296.8 | 3.678  (0.058) |  |
| **4:0 CQ-PQ** | **CQ** | 1072.3 | 45.6 | - | - |
|  | **PQ** | - | - | - |  |
| **0:4 CQ-TQ** | **CQ** | - | - | - | - |
|  | **TQ** | 1232.6 | 78.9 | - |  |
| **1:3 CQ-TQ** | **CQ** | 26.7 | 6.2 | 0.012  (0.001) | 0.9 |
|  | **TQ** | 1068.3 | 21.0 | 0.871  (0.051) |  |
| **1:1 CQ-TQ** | **CQ** | 93.8 | 14.1 | 0.051  (0.002) | 1.3 |
|  | **TQ** | 1477.5 | 70.0 | 1.200  (0.014) |  |
| **3:1 CQ-TQ** | **CQ** | 318.9 | 39.6 | 0.332  (0.003) | 3.0 |
|  | **TQ** | 3186.1 | 38.1 | 2.600  (0.139) |  |
| **4:0 CQ-TQ** | **CQ** | 985.0 | 37.3 | - | - |
|  | **TQ** | - | - | - |  |

^a^ Volume-volume (v/v) mixtures (see Methods).

^b^CQ – chloroquine, AQ – amodiaquine, PQ – primaquine, TQ – tafenoquine, MB – methylene blue.

^c^Result of duplicate experiments, each performed in triplicate (6 determinations total).

^d^S.E.M. – standard error of the mean.

^e^See Equations 1 and 2 in Methods.
