# Supplementary material for: Cytostatic versus cytocidal profiling of quinoline drug combinations via modified fixed-ratio isobologram analysis
Source: Malar J. 2013 Sep 18;12:332. doi: 10.1186/1475-2875-12-332 (PMC3874740; doi:10.1186/1475-2875-12-332)
Supplement: Additional file 6 — PfMDR1 amino acid substitutions associated with CQS and CQR PfMDR1 isoforms in Plasmodium falciparum. [file 1475-2875-12-332-S6.doc]

**Additional File 6.** PfMDR1 amino acid substitutions associated with CQS and CQR PfMDR1 isoforms in *Plasmodiun falciparum*.

|  | | | **PfMDR1 Amino Acid Positions** | | | | |
| --- | --- | --- | --- | --- | --- | --- | --- |
| **Clone/Isolate** | **Origin** | **Classification** | **86** | **184** | **1034** | **1042** | **1246** |
| **HB3**^a^ | Honduras | CQS | N | F | S | D | D |
| **K1**^a^ | S.E. Asia | CQR | Y | Y | S | N | D |
| **FCB**^a^ | Thailand/  S. Africa | CQR | Y | Y | S | N | D |
| **Dd2**^b^ | Indochina | CQR | Y | Y | S | N | D |

^a^Foote, S. J.; Kyle, D. E.; Martin, R. K.; Oduola, A. M.; Forsyth, K.; Kemp, D. J.; Cowman, A. F. *Nature* **1990**, *345*, 255-258.

^b^ Sisowath, C.; Petersen, I.; Veiga, M. I.; Mårtensson, A.; Premji, Z.; Björkman, A.; Fidock, D. A.; Gil, J. P. *J. Infect. Dis.* **2009**, *199*, 750-757.
